# Docosahexaenoic Acid Induces Expression of NAD(P)H: Quinone Oxidoreductase and Heme Oxygenase-1 through Activation of Nrf2 in Cerulein-Stimulated Pancreatic Acinar Cells

**DOI:** 10.3390/antiox9111084

**Published:** 2020-11-04

**Authors:** Yu Jin Ahn, Joo Weon Lim, Hyeyoung Kim

**Affiliations:** Department of Food and Nutrition, BK21 FOUR, College of Human Ecology, Yonsei University, Seoul 03722, Korea; anyujin@gmail.com (Y.J.A.); jwlim11@yonsei.ac.kr (J.W.L.)

**Keywords:** cerulein, docosahexaenoic acid, interleukin 6, nuclear factor erythroid-2-related factor 2, pancreatic acinar cells

## Abstract

Oxidative stress is a major risk factor for acute pancreatitis. Reactive oxygen species (ROS) mediate expression of inflammatory cytokines such as interleukin-6 (IL-6) which reflects the severity of acute pancreatitis. The nuclear factor erythroid-2-related factor 2 (Nrf2) pathway is activated to induce the expression of antioxidant enzymes such as NAD(P)H: quinone oxidoreductase 1 (NQO1) and heme oxygenase-1 (HO-1) as a cytoprotective response to oxidative stress. In addition, binding of Kelch-like ECH-associated protein 1 (Keap1) to Nrf2 promotes degradation of Nrf2. Docosahexaenoic acid (DHA)—an omega-3 fatty acid—exerts anti-inflammatory and antioxidant effects. Oxidized omega-3 fatty acids react with Keap1 to induce Nrf2-regulated gene expression. In this study, we investigated whether DHA reduces ROS levels and inhibits IL-6 expression via Nrf2 signaling in pancreatic acinar (AR42J) cells stimulated with cerulein, as an in vitro model of acute pancreatitis. The cells were pretreated with or without DHA for 1 h and treated with cerulein (10^−8^ M) for 1 (ROS levels, protein levels of NQO1, HO-1, pNrf2, Nrf2, and Keap1), 6 (IL-6 mRNA expression), and 24 h (IL-6 protein level in the medium). Our results showed that DHA upregulates the expression of NQO1 and HO-1 in cerulein-stimulated AR42J cells by promoting phosphorylation and nuclear translocation of Nrf2. DHA increased interaction between Keap1 and Nrf2 in AR42J cells, which may increase Nrf2 activity by inhibiting Keap1-mediated sequestration of Nrf2. In addition, DHA-induced expression of NQO1 and HO-1 is related to reduction of ROS and IL-6 levels in cerulein-stimulated AR42J cells. In conclusion, DHA inhibits ROS-mediated IL-6 expression by upregulating Nrf2-mediated expression of NQO1 and HO-1 in cerulein-stimulated pancreatic acinar cells. DHA may exert positive modulatory effects on acute pancreatitis by inhibiting oxidative stress and inflammatory cytokine production by activating Nrf2 signaling in pancreatic acinar cells.

## 1. Introduction

Acute pancreatitis involves mild and severe inflammatory conditions of the pancreas. Although the pathogenic mechanisms of acute pancreatitis remain poorly understood, oxidative stress and inflammatory mediators are suggested as major factors in the development of acute pancreatitis [1,2]. Chronic pancreatitis often results in chronic abdominal pain and is most commonly caused by excessive alcohol use, smoking, or genetic mutations [3]. Since chronic alcohol abuse promotes generation of reactive oxygen species (ROS) [4] which are responsible for the initiation of the inflammatory process in the pancreatic acinar cells [5], oxidative stress is reported to be involved in the pathogenesis of both acute and chronic pancreatitis.

ROS and reactive nitrogen species (RNS) have been implicated in the pathogenesis of acute pancreatitis. ROS and RNS act directly on biomolecules (lipids, proteins, and nucleic acids) and oxidize these components of cell membrane in the pancreas leading to membrane disintegration and necrosis of the pancreatic cells. In addition, ROS and RNS can also serve as secondary messengers in intracellular signaling and induce pro-inflammatory cascades [6].

Cerulein, a cholecystokinin analogue, has been shown to induce oxidative stress and inflammation in experimental models of acute pancreatitis [7]. Cerulein-induced pancreatitis is characterized by histological changes, increased serum amylase and lipase levels, pro-inflammatory cytokine release, and activation of trypsinogen in acinar cells [8,9,10]. Our previous studies have shown that reactive oxygen species (ROS) can activate redox-sensitive transcription factors nuclear factor-kappa B (NF-κB) and activator protein-1 (AP-1), and inflammatory mediators such as mitogen-activated protein kinases (MAPKs) and janus kinase 2 (JAK2)/signal transducer and activator of transcription 3 (STAT3) in pancreatic acinar cells stimulated with high doses of cerulein. These signaling pathways in turn induce cytokine expression and recruit neutrophils and macrophages to the injured pancreatic tissues [5,11,12]. ROS and cytokines may trigger an inflammatory response in the pancreas. Therefore, prevention of oxidative stress and acinar cell injury during the early phase of acute pancreatitis has been suggested to delay progression to pancreatitis.

Nuclear factor erythroid 2-related factor 2 (Nrf2), a major regulator of cytoprotective responses to oxidative stress [13], regulates the expression of more than 200 genes, including those involved in phase II detoxification and antioxidant gene expression [14]. The key signaling proteins within the Nrf2 pathway bind with small musculoaponeurotic fibrosarcoma (Maf) proteins to the antioxidant response element (ARE) in the regulatory regions of target genes. Kelch-like ECH-associated protein 1 (Keap1) is a cysteine-rich repressor protein that can be modified by various oxidants and electrophiles. Three cysteine residues—C151, C273, and C288—have been shown to alter the conformation of Keap1, resulting in the nuclear translocation of Nrf2 and subsequent target gene expression [15,16]. The binding of Keap1 to Nrf2 promotes ubiquitination and proteasome-dependent degradation of Nrf2. However, inhibition of Nrf2 ubiquitination promotes Nrf2 accumulation and dissociation of newly synthesized Nrf2 protein from Keap1. Thereafter, Nrf2 translocates into the nucleus where it binds to ARE and upregulates the expression of Nrf2 target genes such as NAD(P)H quinone oxidoreductase 1 (NQO1), heme oxygenase 1 (HO-1), glutamate-cysteine ligase (GCL), and glutathione S-transferases (GSTs) [15]. Oxidized omega-3 fatty acids or electrophilic lipid mediators react directly with Keap1 to induce Nrf2-regulated gene expression [17].

NQO1 is a xenobiotic-metabolizing enzyme that catalyzes the oxidation of NAD(P)H into NAD(P) in the presence of various quinones. Decreasing the NAD(P)H/NAD(P) ratio by increased production of NQO1 suppressed cisplatin-induced acute kidney injury through modulation of NOX-derived ROS generation in mice [18]. Increased enzymatic activity of NQO1 upon binding of dunnione—an NQO1 substrate—decreased cellular NADPH levels via modulation of NADPH oxidase (NOX) enzymatic activity in a mouse model of cerulein-induced pancreatic injury [19].

In addition, HO-1 is upregulated by heme, nitric oxide, cytokines, and modified lipids. This cytoprotective enzyme generates iron ions, biliverdin, and CO. Biliverdin, together with bilirubin, acts as an antioxidant. Moreover, the other products of HO-1 can mediate important biological processes. Therefore, HO-1 exerts cytoprotective effects that are activated by inflammation, hypoxia, hyperoxia, or radiation [20,21]. Since ROS mediate cytokine expression to propagate AP, reducing ROS by antioxidant enzymes such as NQO1 and HO-1 may attenuate the development of AP.

Previous studies showed that upregulation of HO-1 or treatment with its downstream effectors and heme degradation products, biliverdin and CO, have protective effects in different rodent models of acute pancreatitis induced by taurocholate, cerulein, or choline-deficient, ethionine-supplemented diet [22,23,24,25,26,27]. HO-1 overexpressing macrophages protect against acute pancreatitis [22,28]. Fusco et al. [29] reported that hydroxytyrosol inhibited cerulein-induced acute pancreatitis by enhancing expression of Nrf2 and HO-1 in mice. Naringenin has antioxidant properties and protects against acute pancreatitis in two experimental models (cerulein-induced mild model and arginine-induced severe model) in mice by Nrf2/HO-1 pathways [30]. Isoliquiritigenin, a flavonoid monomer with confirmed antioxidant activity, ameliorates acute pancreatitis in mice via inhibition of oxidative stress and modulation of the Nrf2/HO-1 pathway [31]. High-dose vitamin C alleviates pancreatic injury via the NRF2/NQO1/HO-1 pathway in a rat model of severe acute pancreatitis [32]. Kambhampati et al. [33] suggested that HO-1 and its downstream effectors are potential targets for clinical acute pancreatitis. They demonstrated that antisecretory agents, protease inhibitors, immunomodulators and anti-inflammatory agents, and antioxidants may be promising therapeutic agents. However, these therapies still remain inconclusive and have not been translated into current standard treatment care, yet.

Docosahexaenoic acid (DHA, 22:6n-3)—an omega-3 polyunsaturated fatty acid—exhibits antioxidative and anti-inflammatory properties in various disease models [34]. We reported that DHA activated peroxisome proliferator-activated receptor γ (PPARγ) and induced catalase to reduce ROS-mediated expression of cytokines in pancreatic acinar cells [35]. In cerulein-induced acute pancreatitis, DHA suppresses IL-1β and IL-6 gene expression by inhibiting AP-1 activation [36]. We summarized that DHA suppressed the expression of inflammatory mediators by inhibiting ROS-mediated activation of PKC-δ, NF-κB, AP-1, JAK2/STAT3, and inflammatory cytokines in a cerulein-stimulated experimental model for acute pancreatitis [37]. Even though there have been no studies for the effect of DHA on Nrf2 activation and induction of antioxidant enzyme induction in pancreatic acinar cells, we postulate that DHA may induce Nrf2-target antioxidant enzymes such as NQO1 and HO-1 in pancreatic acinar cells to reduce ROS levels as a cytoprotective mechanism against oxidative stress including cerulein stimulation.

The purpose of the present study was to examine the effect of DHA on Nrf2 activation and induction of NQO1 and HO-1 in pancreatic acinar cells. We investigated the mechanism by which DHA suppresses ROS-mediated expression of pro-inflammatory cytokine IL-6 in cerulein-stimulated AR42J cells which has been used as an in vitro model of acute pancreatitis. Our data showed experimental evidence for DHA-induced reduction in ROS and IL-6 expression, and upregulation of NQO1 and HO-1 via Nrf2 activation in cerulein-stimulated AR42J cells.

## 2. Materials and Methods

### 2.1. Materials

DHA, trigonelline—an Nrf2 inhibitor—and cerulein were purchased from Sigma-Aldrich (St. Louis, MO, USA). DHA was dissolved in ethanol (0.5 M solution). Trigonelline was dissolved in distilled water (final concentration, 5 mM), while cerulein was dissolved in phosphate-buffered saline (PBS) containing 0.1% bovine serum albumin (BSA) (final concentration, 10^−4^ M). Protoporphyrin (ZnPP)—a HO-1 inhibitor—was purchased from Santa Cruz Biotechnology (sc-691550, Santa Cruz, CA, USA) and dissolved in dimethylsulfoxide (DMSO) (final concentration, 1 mM).

### 2.2. Cell Line and Culture Conditions

Rat pancreatic acinar AR42J cells (pancreatoma, ATCC CRL 1492) were obtained from the American Type Culture Collection (ATCC; Manassas, VA, USA) and cultured in Dulbecco’s modified Eagle’s medium (Sigma, St. Louis, MO, USA) supplemented with 10% fetal bovine serum (GIBCO-BRL, Grand Island, NY, USA) and antibiotics (100 U/mL penicillin and 100 μg/mL streptomycin). Cells were cultured at 37 °C in a humidified atmosphere of 95% air and 5% CO_2_.

### 2.3. Experimental Protocol

To determine the effect of DHA in unstimulated cells, AR42J cells (8 × 10^5^/dish) were treated with DHA (50 μM) for 1, 2, and 3 h and the expression of Keap1, NQO1, and HO-1, and phosphorylated and total Nrf2 level were assessed in whole-cell extracts or nuclear extracts. To study the dissociation of the Keap1-Nrf2 complex, cells (8 × 10^5^/dish) were treated with DHA (50 μM) for 1 h, and interaction between Nrf2 and Keap1 was determined in whole-cell extracts and whole-cell extract-derived immunoprecipitates obtained using the anti-Nrf2 and anti-Keap 1 antibodies for precipitation. To observe nuclear translocation of Nrf2, AR42J cells (2 × 10^5^/well) treated with 50 μM DHA for 1 h and confocal microscope images were obtained by immunofluorescence staining of the fixed cells.

Prior to the experiments on the effect of DHA on mRNA and protein expression of IL-6 and ROS levels in cerulein-stimulate cells, time-course experiments were performed to determine effective stimulation time for increases in IL-6 expression and ROS levels. Cells (2 × 10^5^/well) were stimulated with cerulein (10^−8^ M) for 0.25, 0.5, 1, 2, 4, 6, 8, and 24 h. Cerulein stimulated IL-6 expression in AR42J cells; mRNA expression of IL-6 peaked at 6 h and maintained until 24 h while protein levels of IL-6 peaked at 24 h. ROS levels were increased by cerulein in AR42J cells and peaked at 1 h and maintained until 24 h. Therefore, to determine the effect of DHA on cerulein-induced increases in the levels of IL-6 (mRNA and protein) and ROS, the cells were pretreated with DHA for 1 h and treated with cerulein for 6 (for determination of IL-6 mRNA level), 24 (for determination of IL-6 protein level), or 1 h (for determination of ROS levels).

To determine the effect of DHA on IL-6 mRNA and ROS levels in unstimulated cells (cells without cerulein stimulation), cells (2 × 10^5^/well) were treated with DHA alone for 2 (ROS levels) and 7 h (for IL-6 mRNA level), respectively. This is because DHA was pre-treated for 1 h and then stimulated with cerulein for 6 (IL-6 mRNA) and 1 h (for ROS) to assess the effect of DHA on cerulein-stimulated increases in IL-6 and ROS levels.

To determine the effect of DHA on IL-6, NQO1, and HO-1 expression as well as ROS levels in cerulein-stimulated cells, AR42J cells were treated with DHA (20 or 50 μM) for 1 h and stimulated with cerulein (10^−8^ M) for 1 (for NQO1, HO-1, and ROS), 6 (for IL-6 mRNA), and 24 h (for IL-6 protein in the medium).

To assess whether Nrf2, NQO1, and HO-1 contribute to the inhibitory effect of DHA on cerulein-stimulated IL-6 expression, cells were pretreated with trigonelline (5 μM) or ZnPP (1 μM) in the presence of DHA (50 μM) for 1 h and stimulated with cerulein for 1 (for NQO1, HO-1, and ROS), 6 (for IL-6 mRNA), and 24 h (for IL-6 protein).

To determine the effect of trigonellin alone or ZnPP alone for IL-6 mRNA expression and ROS levels in unstimulated cells (cells without cerulein stimulation), the cells were treated with trigonelline (5 μM) or ZnPP (1 μM) for 2 (ROS levels) and 7 h (for IL-6 mRNA level).

Regarding cell numbers, cells (8.0 × 10^5^/dish) were pretreated with or without DHA, trigonelline, or ZnPP for 1 h and treated with or without cerulein (10^−8^ M) for 1 h to determine protein levels of NQO1, HO-1, pNrf2, Nrf2, and Keap1 in whole cell extracts or nuclear extracts by Western blotting. For the determination of nuclear translocation of Nrf2 (by immunofluorescence staining), IL-6 mRNA expression (real-time PCR), IL-6 protein level in the medium (enzyme-linked immunosorbent assay), and ROS levels (fluorescence assay), cells (2.0 × 10^5^/well) were pretreated with or without DHA inhibitors, trigonelline, or ZnPP for 1 h and treated with cerulein (10^∔8^ M) for 1 (ROS levels, nuclear translocation of Nrf2 by immunofluorescence staining), 6 (IL-6 mRNA expression), and 24 h (IL-6 protein level in the medium).

### 2.4. Determination of Intracellular ROS Levels

For time-course experiments, the cells (2.0 × 10^5^/well) were stimulated with cerulein for 0.25, 1, 2, 4, 6, 8, and 24 h. For the effect of DHA alone, trigonelline alone, or ZnPP alone on ROS levels in unstimulated cells, cells (2.0 × 10^5^/well) were treated with or DHA, trigonelline or ZnPP for 2 h. To determine the effect of DHA, trigonelline, or ZnPP on cerulein-induced increases in ROS, cells (2.0 × 10^5^/well) were pre-treated with DHA, trigonelline, or ZnPP for 1 h and treated with cerulein for 1 h. For determination of ROS, the cells were incubated with 10 μg/mL dichlorofluorescein diacetate (DCF-DA; Sigma-Aldrich) for 30 min at 37 °C. Cells were washed twice with PBS and the fluorescence intensity DCF was measured at 535 nm (excitation at 495 nm) using a Victor 5 multi-label counter (PerkinElmer Life and Analytical Sciences, Boston, MA, USA). Intracellular ROS levels were normalized to cell numbers and expressed as the relative percentage of controls.

### 2.5. Real-Time PCR

Total RNA was isolated using TRI reagent (Molecular Research Center, Cincinnati, OH, USA) and reverse-transcribed into cDNA using a random hexamer and M-MLV reverse transcriptase (Promega, Madison, WI, USA) with conditions at 23 °C for 10 min, 37 °C for 60 min, and 95 °C for 5 min. The cDNA was incubated with SYBR Green Real-time PCR Master Mix (Toyobo, Osaka, Japan) that contained 10 pg/mL of forward and reverse primers, and amplified using a Light Cycler PCR system (Roche Applied Sciences, Indianapolis, IN, USA). Real-time PCR was conducted with the following rat-specific primers for IL-6 and β-actin. The IL-6 (accession number NM_012589) primers 5′-GAGAGGAGACTTCACAGAGGATACCA-3′ (forward primer) and 5′-CCACAGTGAGGAATGTCCACA-3′ (reverse primer) were used to generate a 590 bp PCR product. For β-actin, the forward primer used is 5′-ACCAACTGGGACATGGAG-3′ and the reverse primer used is 5′-GTCACGATCTTCATGAGGTAGTC-3′, giving an 890 bp PCR product. For PCR amplification, the cDNA was amplified by 40 repeat denaturation cycles at 95 °C for 30 s, annealing at 54 °C for 30 s, and extension at 72 °C for 45 s. During the first cycle, the 95 °C step was extended to 3 min. The β-actin gene was amplified in the same reaction as a reference gene.

### 2.6. Enzyme-Linked Immunosorbent Assay

AR42J cells, 2.0 × 10^5^/well, were pretreated with or without DHA, trigonelline, or ZnPP for 1 h and treated with cerulein for 24 h. The culture medium was centrifuged at 15,000× *g* for 15 min at 4 °C. The supernatant was collected to determine IL-6 concentration using an enzyme-linked immunosorbent assay (ELISA) kit (Invitrogen Corporation, Carlsbad, CA, USA), following the manufacturer’s instructions.

### 2.7. Preparation of Whole-Cell and Nuclear Extracts

Cells were harvested by scraping in PBS and centrifugation at 5000× *g* for 15 min. The cells were resuspended in lysis buffer containing 10 mM Tris (pH 7.4), 1% NP-40, and protease inhibitor (Complete; Roche, Mannheim, Germany), and lysed by passing them through a 1-mL syringe. The lysate was incubated on ice for 30 min and centrifuged at 13,000× *g* for 15 min. The supernatants were collected as whole cell extracts. The cytosolic and nuclear extracts were prepared using a NE-PER^®^ nuclear and cytoplasmic extraction reagent kit (Thermo Fisher, Waltham, MA, USA) according to the manufacturer’s instructions. In brief, cells were suspended in cytoplasmic extraction reagent containing protease inhibitor and vortexed for 15 s followed by centrifugation at 16,000× *g* for 5 min. The supernatants were cytosolic extracts. The pellets were resuspended in nuclear extraction reagent, vortexed, and centrifuged at 16,000× *g* for 10 min. The supernatants were collected and used as the nuclear extracts. The specificity of the nuclear extracts was confirmed by the presence of lamin A/C predominantly in the nuclear fraction. Bradford assay (Bio-Rad Laboratories, Hercules, CA, USA) was used for the determination of protein concentration.

### 2.8. Western Blotting

Whole-cell extracts (6–40 μg/per lane) were loaded onto 8–10% SDS polyacrylamide gels and separated by electrophoresis under reducing conditions. The proteins were then transferred to nitrocellulose membranes (Amersham, Inc., Arlington Heights, IL, USA) by electroblotting and verified using reversible staining with Ponceau S. The membranes were blocked with 3% non-fat dry milk in Tris-buffered saline and 0.2% Tween 20 (TBS-T). Proteins were detected with antibodies against Nrf2 (ab62352, Abcam, Cambridge, UK), p-Nrf2 (ab76026, Abcam), Keap1 (sc-365626, Santa Cruz Biotechnology), HO-1 (ADI-SPA-895, Enzo Life Science Inc., Farmingdale, NY, USA), NQO1 (ab2346, Abcam), lamin A/C (4777S, Cell Signaling, MA, USA), and actin (sc-1615, Santa Cruz Biotechnology) in TBS-T containing 3% non-fat dry milk and incubated overnight at 4 °C. After washing with TBS-T, primary antibodies were detected with horseradish peroxidase-conjugated secondary antibodies (anti-rabbit, anti-mouse, anti-goat) and visualized using the enhanced chemiluminescence detection system (Santa Cruz Biotechnology, Dallas, TX, USA) and BioMax MR film (Kodak, Rochester, NY, USA) using the enhanced chemiluminescence detection system (Santa Cruz Biotechnology). Actin and lamin A/C served as loading controls in whole-cell extracts and nuclear extracts.). Protein level was compared to that of the loading control actin. Intensity of each protein band was densitometrically quantified by using the software Image J (National Institutes of Health, USA). The densitometry data represent means ± S.E. from three immunoblots and are shown as relative density of protein band normalized to actin level.

### 2.9. Immunoprecipitation of the Nrf2-KEAP 1 Complex

DHA (50 μM)-treated and untreated AR42J cells were lysed in 500 μM immunoprecipitation buffer containing 10 mM Tris-HCl (pH 7.4), 100 mM NaCl, 1 mM EDTA, 1 mM EGTA, Complete, 0.5% NP-40, 0.5% sodium deoxycholate, and 10% glycerol. Then, cells were centrifuged at 15,000× *g* for 15 min. The polyclonal antibody and protein G-agarose were added to the cleared supernatant, and the mixture was incubated overnight at 4 °C. The protein G-antibody-antigen complex was collected by washing four times with immunoprecipitation buffer containing 150 mM NaCl, 10 mM Tris-HCl (pH 7.4), 1 mM EDTA, 1 mM EGTA, 0.5% NP-40, and 0.5% sodium deoxycholate. The pellet was resuspended in 50 μL SDS sample buffer and boiled for 10 min. The preparation was subsequently subjected to Western blotting.

### 2.10. Immunofluorescence Staining

Cells (2 × 10^5^/well) were treated with DHA for 1 h on glass slides and fixed with cold 100% methanol. The fixed cells were permeabilized with 0.1% Triton X-100 in PBS for 5 min, blocked with 0.1% gelatin and 1% bovine serum albumin in PBS for 1 h, and incubated with the primary antibody against Nrf2 for 1 h. After washing with PBS, the cells were incubated with rhodamine-conjugated mouse anti-rabbit IgG antibody (sc-2492, Santa Cruz Biotechnology) for 1 h. After removal of the secondary antibody, the cells were washed with PBS and coverslipped with antifade medium Vectashield containing 4′,6-diamidino-2-phenylindole (DAPI). The slides were incubated for 30 min to allow saturation with DAPI. Cells stained with the rhodamine-conjugated antibody were examined under a laser scanning confocal microscope (Zeiss LSM 880, Carl Zeiss Inc., Thornwood, NY, USA), and images were acquired. The intensity of NRF2 red fluorescence in the nuclei was quantified. Values represent means ± S.E. from three independent experiments.

### 2.11. Statistical Analysis

All values are expressed as the mean ± standard error (SE) from three different experiments. For each experiment, the number of each group was 4 (*n* = 4 per each group). Analysis of variance (ANOVA), followed by the Newman–Keul post hoc test, was used for the statistical analysis. A *p*-value of 0.05 or less was considered statistically significant.

## 3. Results

### 3.1. DHA Increases Expression, Phosphorylation, and Nuclear Translocation of Nrf2, and Expression of NQO1 and HO-1 in Unstimulated AR42J Cells

**Figure 1:**
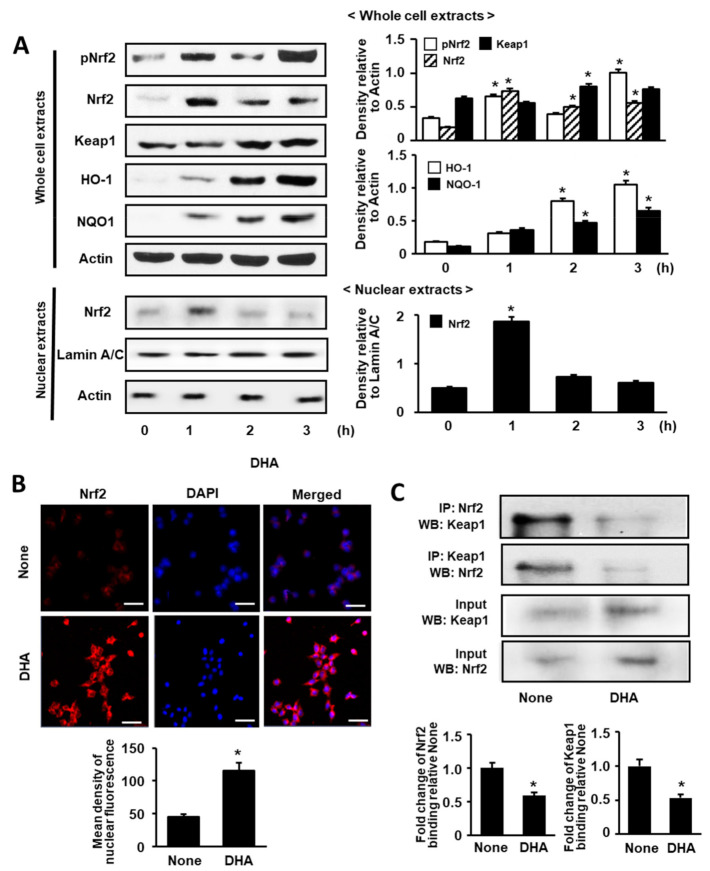
Effect of DHA on the levels of activated Nrf2, Nrf2, Keap 1, HO-1, NQO1, and Keap 1-bound Nrf2 in AR42J cells. (**A**) Cells (8.0 × 10^5^/dish) were treated with 50 μM DHA for the indicated time periods. Protein levels were determined by Western blot analysis in whole-cell extracts (upper panel) or nuclear extracts (lower panel) with actin as the loading control and lamin A/C as the index for the nuclear extracts. The densitometry data represent means ± S.E. from three immunoblots and are shown as relative density of protein band normalized to actin or lamin A/C level. (**B**) Nuclear translocation of Nrf2 was determined with confocal microscope images of AR42J cells (2 × 10^5^/well) treated with 50 μM DHA for 1 h followed by immunofluorescence staining of the fixed cells (upper panel). Nrf2 was visualized using fluorescein/rhodamine-conjugated anti-rabbit IgG antibody (red) with DAPI counter staining (blue) of the same field. “None” refers to cells treated with the vehicle for DHA (0.5 M ethanol) only. The scale bar was 50 μm. The intensity of NRF2 red fluorescence in the nuclei was quantified (lower panel). Values represent the means of three independent experiments. (**C**) Cells (8 × 10^5^/dish) were treated with 50 μM DHA for 1 h. Interaction between Nrf2 and Keap1 was determined by Western blots of whole-cell extracts and whole-cell extract-derived immunoprecipitates obtained using the anti-Nrf2 and anti-Keap 1 antibodies for precipitation (IP) and visualization (WB; Western blot analysis), as indicated (upper panel). Input is used as the control for protein expression. “None” refers to cells treated with the vehicle for DHA (0.5 M ethanol) only. Quantification of binding of Nrf2 to keap1 or binding of Keap1 to Nrf2 is based on band intensity of Western blot analysis using Image J software (lower panel). The densitometry data represent means ± S.E. from three immunoblots. * *p* < 0.05 vs. none.

We first determined the effect of DHA in unstimulated cells by measuring DHA-induced changes in Nrf2 expression, phosphorylation, and nuclear translocation. Subsequently, whole-cell extracts and nuclear extracts of AR42J cells treated with DHA for 1, 2, and 3 h were subjected to Western blot analysis. As shown in Figure 1A, Nrf2 expression in whole-cell and nuclear extracts peaked at 1 h. However, p-Nrf2 expression in the whole-cell extracts reached the maximum at 1 and 3 h. (Figure 1A). Actin, the loading control, and lamin A/C, the index of nuclear extracts, were not changed by DHA treatment. Lamins are nuclear membrane structural components that are important in maintaining normal cell functions. Lamin A/C antibody detects endogenous levels of total full length lamin A (and lamin C) (70 kDa). Therefore, we used lamin A/C as the index of nuclear extracts. To determine the effect of DHA on nuclear translocation of Nrf2, AR42J cells were incubated with DHA for 1 h and then subjected to immunofluorescence staining. The expression level of DAPI, the nuclear marker, was maintained; however, nuclear expression of Nrf2, with red immunofluorescence staining, was increased (Figure 1B).

Next, we examined the effect of DHA on Keap1, NQO1, and HO-1 expression by Western blot analysis of whole-cell extracts. As shown in Figure 1A, the levels of Keap1, NQO1, and HO-1 were increased in a time-dependent manner over a 3-h period. These results indicate that DHA activates Nrf2 and upregulates NQO1 and HO-1 expression. Nrf2 dissociates from Keap1 to regulate oxidative stress; therefore, we carried out an additional experiment (see below) to determine whether treatment with DHA can affect the level of the Nrf2-Keap1 complex.

### 3.2. DHA Decreases Interaction between Keap1 and Nrf2 in AR42J Cells

To validate the interaction between Keap1 and Nrf2 in AR42J cells, we performed immunoprecipitation (IP) followed by Western blot (WB) analysis using the anti-Nrf2 antibody and anti-Keap1 antibody, respectively.

As shown in Figure 1C, cells were treated with DHA (50 μM) for 1 h or left untreated (“None”). The protein levels of Keap1 and Nrf2 in whole-cell extracts of DHA-treated cells increased (Figure 1C, lower panel), whereas that in immunoprecipitated fraction decreased (Figure 1C, upper panel). This result indicates that DHA may increase Nrf2 activity by inhibiting Keap1-mediated sequestration of Nrf2.

### 3.3. Cerulein Increases IL-6 Expression and ROS Levels, but DHA Alone Did Not Increasse IL-6 mRNA and ROS Levels in AR42J Cells

In eucaryotic cells, ROS are produced in the reactions catalyzed by NAD(P)H oxidase and by some other specialized oxidases and also as a byproduct of many redox reactions. ROS act as secondary messengers responsible for a signal transduction from extracellular signaling molecules and their membrane receptors to the intracellular regulatory systems which control gene expression [38]. Therefore, ROS production is an early event, followed by mRNA expression and later protein expression.

**Figure 2:**
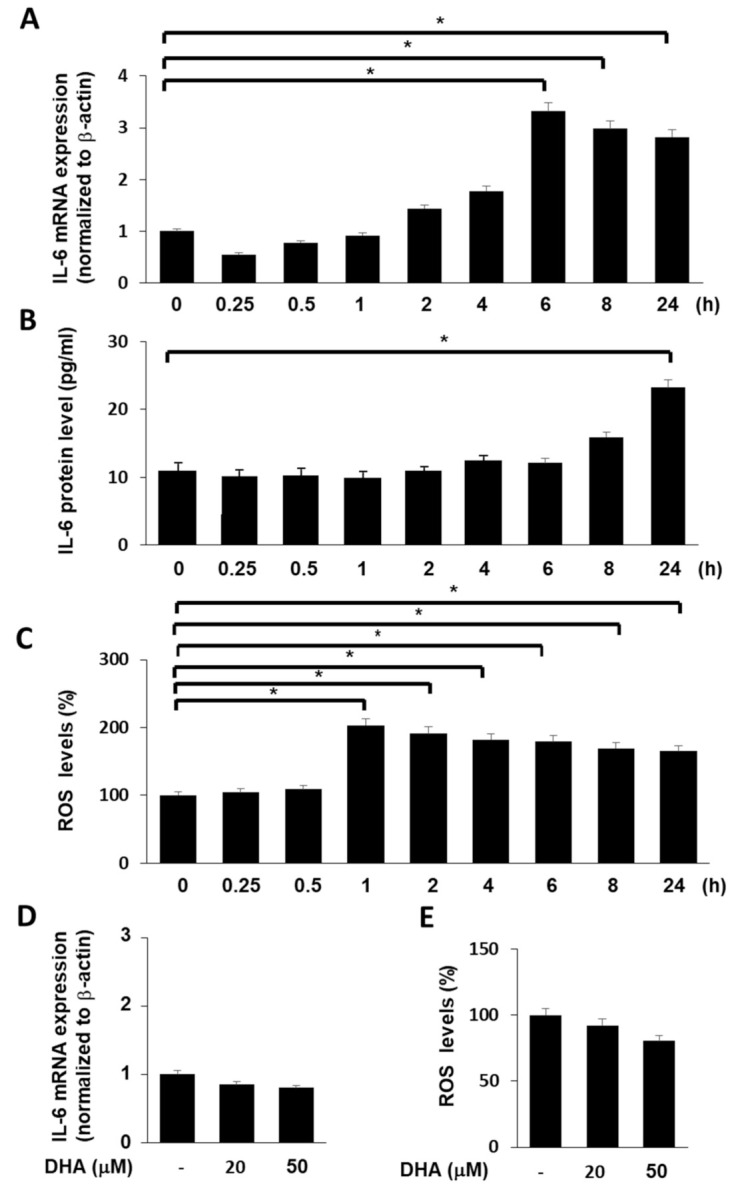
Effect of cerulein or DHA on the expression of IL-6 and ROS levels in AR42J cells. (**A**) Cells (2 × 10^5^/well) were stimulated with cerulein (10^−8^ M) for the indicated time periods (0.25, 0.5, 1, 2, 4, 6, 8, and 24 h). IL-6 mRNA expression was determined by real-time PCR analysis and normalized to β-actin mRNA expression. (**B**) Cells (2 × 10^5^/well) were stimulated with cerulein (10^−8^ M) for the indicated time periods (0.25, 0.5, 1, 2, 4, 6, 8, and 24 h). IL-6 concentration in the cell culture medium was determined by ELISA. (**C**) Cells (2 × 10^5^/well) were stimulated with cerulein (10^−8^ M) for the indicated time periods (0.25, 0.5, 1, 2, 4, 6, 8, and 24 h). Then, cells were incubated with 10 μg/mL dichlorofluorescein diacetate (DCF-DA) for 30 min and washed with PBS. Cellular ROS levels were determined by measuring the level of fluorescent DCF. Intracellular ROS levels are expressed as the relative increase. The value for ROS levels in unstimulated cells (cells without cerulein stimulation) was set at 100%. Data are expressed as the mean ± S.E. of three independent experiments. * *p* < 0.05. (**D**) Cells (2 × 10^5^/well) were treated with DHA (20 or 50 μM) for 7 h. IL-6 mRNA expression was determined by real-time PCR analysis and normalized to β-actin mRNA expression. (E) Cells (2 × 10^5^/well) were treated with DHA (20 or 50 μM) for 2 h. Then, cells were incubated with 10 μg/mL DCF-DA for 30 min and washed with PBS. Cellular ROS levels were determined by measuring the level of fluorescent DCF. Intracellular ROS levels are expressed as the relative increase. The value for ROS levels in cells in the absence of DHA was set at 100%. Data are expressed as the mean ± S.E. of three independent experiments. * *p* < 0.05. −, without treatment.

Prior to the experiments on the effect of DHA on mRNA and protein expression of IL-6 and ROS levels in cerulein-stimulate cells, time-course experiments were performed. Cells (2 × 10^5^/well) were stimulated with cerulein (10^−8^ M) for 0.25, 0.5, 1, 2, 4, 6, 8, and 24 h. Figure 2A shows that cerulein increased IL-6 mRNA expression. mRNA expression of IL-6 peaked at 6 h and maintained until 24 h. Protein levels of IL-6 were increased by cerulein 1(Figure 2B). Protein levels of IL-6 peaked at 24 h. ROS levels were increased by cerulein in AR42J cells and peaked at 1 h (Figure 2C). Thus, for the further studies on the effect of DHA on cerulein-induced increases in the levels of IL-6 (mRNA and protein) and ROS, the cells were pretreated with DHA for 1 h treated with cerulein for 6 (for determination of IL-6 mRNA level), 24 (for determination of IL-6 protein level), or 1 h (for determination of ROS levels).

To determine the effect of DHA alone on IL-6 mRNA and ROS levels in unstimulated cells (cells without cerulein stimulation), the cells were treated with DHA for 7 (for IL-6 mRNA) and 2 h (for ROS levels), respectively. DHA did not increase IL-6 mRNA and ROS levels in AR42J cells (Figure 2D,E).

### 3.4. DHA Upregulates Expression of NQO1 and HO-1 in Cerulein-Stimulated AR42J Cells

**Figure 3:**
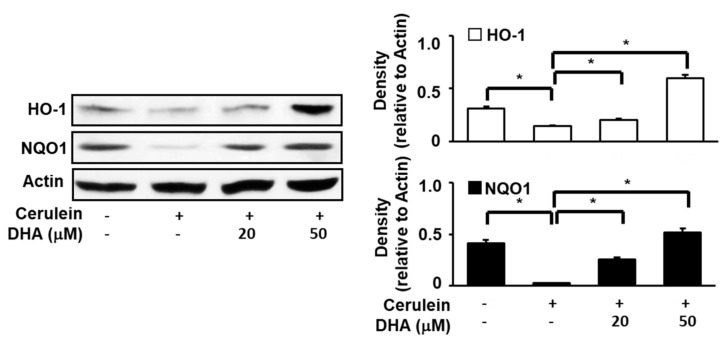
Effect of DHA on the expression of HO-1 and NQO1 in cerulein-stimulated AR42J cells. Cells (8 × 10^5^/dish) were pretreated with DHA (20 or 50 μM) for 1 h and subsequently stimulated with cerulein (10^−8^ M) for 1 h. Protein levels of HO-1 and NQO1 in whole-cell extracts were determined by Western blot analysis, using actin as the loading control. The densitometry data represent means ± S.E. from three immunoblots and are shown as relative density of protein band normalized to actin level. * *p* < 0.05. +, with treatment; −, without treatment.

To determine the effect of DHA on the expression of HO-1 and NQO1 in cerulein-stimulated AR42J cells, cells were pretreated with DHA (20 or 50 μM) for 1 h and subsequently stimulated with cerulein (10^−8^ M) for 1 h. As shown in Figure 3, the expression of Nrf2 target genes such as *NQO1* and *HO-1* was reduced by cerulein. However, pretreatment with DHA induced a net increase in both NQO1 and HO-1 expression. These findings suggest that DHA increases the expression of antioxidant genes, *NQO1* and *HO-1*, which may reduce oxidative stress in cerulein-stimulated AR42J cells.

### 3.5. Nrf2 Inhibitor Trigonelline Suppresses the Effect of DHA on Expression of IL-6, NQO1, and HO-1 and ROS Levels in Cerulein-Stimulated AR42J Cells

**Figure 4:**
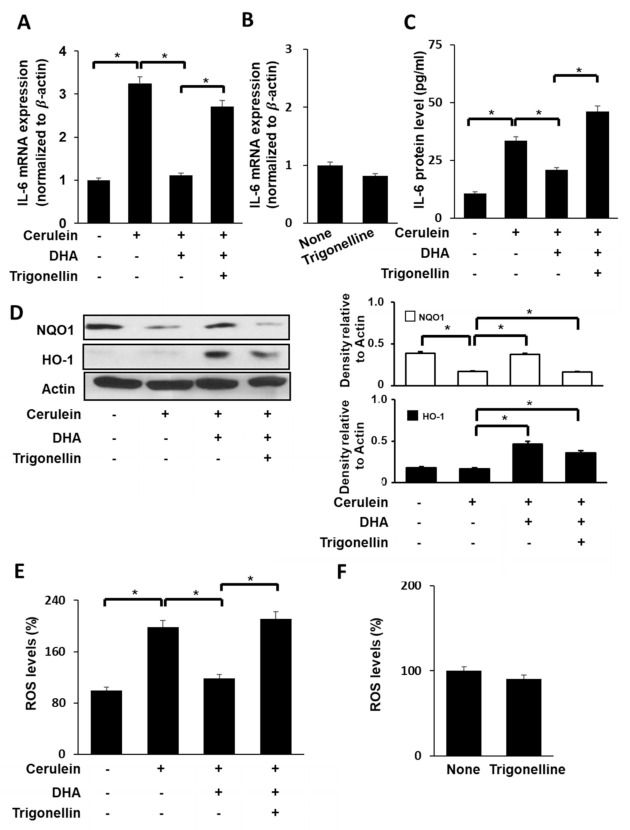
Effect of Nrf2 inhibitor trigonelline on the expression of IL-6, NQO1, and HO-1, and ROS levels in cerulein-stimulated DHA-treated AR42J cells. (**A**) Cells (2 × 10^5^/well) were pretreated with trigonelline (5 μM) in the presence of DHA (50 μM) for 1 h, and stimulated with cerulein for 6 h. IL-6 mRNA expression was determined by real-time PCR analysis and normalized to β-actin mRNA expression. (**B**) Cells (2 × 10^5^/well) were treated with trigonelline (5 μM) alone for 7 h. IL-6 mRNA expression was determined by real-time PCR analysis and normalized to β-actin mRNA expression. (**C**) Cells (2 × 10^5^/well) were pretreated with trigonelline (5 μM) in the presence of DHA (50 μM) for 1 h and stimulated with cerulein for 24 h. IL-6 concentration in the culture medium was determined using ELISA. (**D**) Cells (8 × 10^5^/dish) were pretreated with trigonelline (5 μM) in the presence of DHA (50 μM) for 1 h and stimulated with cerulein for 1 h. Protein levels of HO-1 and NQO1 in whole-cell extracts were determined by Western blot analysis, using actin as the loading control (right panel). The densitometry data represent means ± S.E. from three immunoblots and are shown as relative density of protein bands normalized to actin level (left panel). (**E**) Cells (2 × 10^5^/well) were treated with trigonelline (5 μM) in the presence of DHA (50 μM) for 1 h and stimulated with cerulein for 1 h. Then, cells were incubated with 10 μg/mL DCF-DA for 30 min and washed with PBS. Cellular ROS levels were determined by measuring the level of fluorescent DCF. (**F**) Cells (2 × 10^5^/well) were treated with trigonelline alone (5 μM) for 2 h. Then, cells were incubated with 10 μg/mL DCF-DA for 30 min and washed with PBS. Cellular ROS levels were determined by measuring the level of fluorescent DCF. Intracellular ROS levels are expressed as the relative increase. The value for ROS levels in unstimulated cells (cells without cerulein) in the absence of DHA was set as 100%. Data are expressed as the mean ± S.E. of three independent experiments. * *p* < 0.05. +, with treatment; −, without treatment.

To determine the effect of DHA on ROS and IL-6 levels in cerulein-stimulated cells, AR42J cells were treated with DHA (50 μM) for 1 h and stimulated with cerulein (10^−8^ M) for 1 (for ROS, Figure 4E, 3rd lane), 6 (for IL-6 mRNA, Figure 4A, 3rd lane), and 24 h (for IL-6 protein in the medium, Figure 4C, 3rd lane). DHA inhibited cerulein-induced increases in ROS and IL-6 mRNA and protein levels.

To assess whether Nrf2, NQO1, and HO-1 contribute to the inhibitory effect of DHA on cerulein-stimulated IL-6 expression, cells were pretreated with a Nrf2 inhibitor trigonelline (5 μM) in the presence of DHA (50 μM) for 1 h and stimulated with cerulein for 1 (for NQO1, HO-1, and ROS), 6 (for IL-6 mRNA), and 24 h (for IL-6 protein).

As shown in Figure 4A,C, trigonelline reversed the inhibitory effect of DHA on IL-6 expression at both mRNA level (Figure 4A) and protein level (Figure 4C) in cerulein-stimulated cells. Treatment of cells with trigonelline decreased the DHA-induced increase in NQO1 and HO-1 expression (Figure 4D) in cerulein-stimulated AR42J cells. The inhibitory effect of DHA on ROS production was reversed by trigonelline treatment in cerulein-stimulated cells (Figure 4E). However, trigonelline alone did not change mRNA expression of IL-6 (Figure 4B) and ROS levels (Figure 4F) in unstimulated cells.

### 3.6. HO-1 Iinhibitor ZnPP Inhibits the Effect of DHA on ROS Levels and IL-6 Expression in Cerulein-Stimulated AR42J Cells

**Figure 5:**
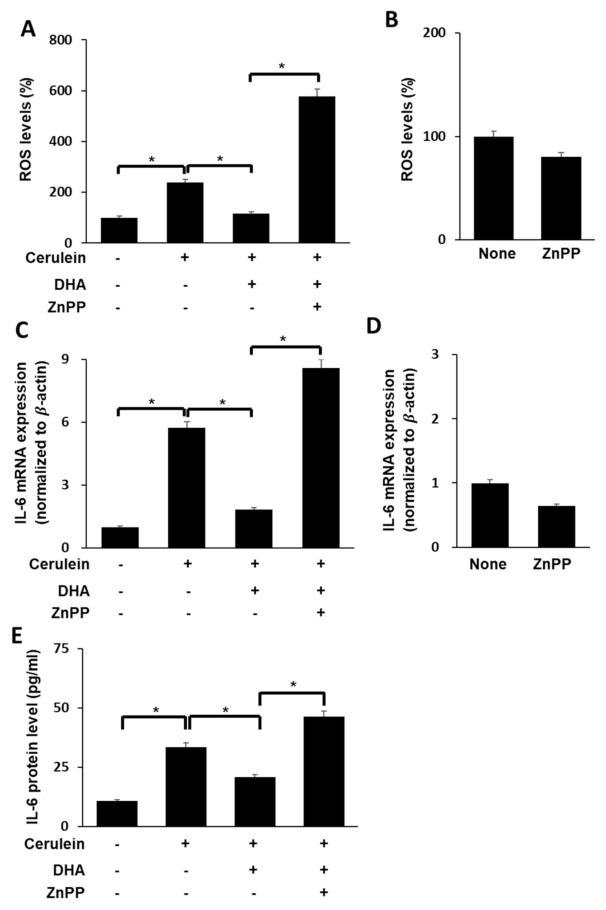
Effect of HO-1 inhibitor ZnPP on ROS levels and IL-6 expression in cerulein-stimulated DHA-treated AR42J cells. (**A**) Cells (2 × 10^5^/well) were treated with ZnPP (1 μM) in the presence of DHA (50 μM) for 1 h and stimulated with cerulein for 1 h. Then, cells were incubated with 10 μg/mL DCF-DA for 30 min and washed with PBS. Cellular ROS levels were determined by measuring the level of fluorescent DCF. (**B**) Cells (2 × 10^5^/well) were treated with ZnPP alone (1 μM) for 2 h. Then, cells were incubated with 10 μg/mL DCF-DA for 30 min and washed with PBS. Cellular ROS levels were determined by measuring the level of fluorescent DCF. Intracellular ROS levels are expressed as the relative increase. The value for ROS levels in unstimulated cells (without cerulein stimulation) in the absence of DHA was set at 100%; (**C**) cells (2 × 10^5^/well) were treated with ZnPP (1 μM) in the presence of DHA (50 μM) for 1 h and stimulated with cerulein for 6 h. IL-6 mRNA expression was determined by real-time PCR analysis and normalized to β-actin mRNA expression. (**D**) Cells (2 × 10^5^/well) were treated with ZnPP alone (1 μM) for 7 h. IL-6 mRNA expression was determined by real-time PCR analysis and normalized to β−actin mRNA expression. (**E**) Cells (2 × 10^5^/well) were treated with ZnPP (1 μM) in the presence of DHA (50 μM) for 1 h and stimulated with cerulein for 24 h. IL-6 concentration in the cell culture medium was determined using ELISA. Data are expressed as the mean ± S.E. of three independent experiments. * *p* < 0.05. +, with treatment; −, without treatment.

We sought to determine whether the DHA-induced decrease in ROS levels and IL-6 expression in cerulein-stimulated cells is mediated by concomitant increase in HO-1 expression. Cerulein-stimulated AR42J cells were treated with DHA in the presence or absence of the HO-1 inhibitor ZnPP. As shown in Figure 5, DHA decreased cerulein-induced increase in ROS levels (Figure 5A) as well as IL-6 expression at both the mRNA (Figure 5C) and protein (Figure 5E) levels. The effect of DHA on IL-6 expression and ROS levels was reversed by ZnPP treatment in cerulein-stimulated cells. These findings suggest that DHA downregulates IL-6 mRNA expression and reduces ROS levels via upregulation of Nrf2 target gene *HO-1* in cerulein-stimulated AR42J cells. However, ZnPP alone did not change ROS levels (Figure 5B) or expression of IL-6 mRNA (Figure 5D) in unstimulated cells.

## 4. Discussion

The present study demonstrated that DHA attenuates cerulein-stimulated increases in ROS and IL-6 expression in pancreatic acinar AR42J cells by upregulating antioxidant enzymes NQO1 and HO-1 through Nrf2 activation. Cerulein activates NADPH oxidase and produces large amounts of ROS which activate oxidant-sensitive transcription factors such as NF-kB to induce IL-6 expression in pancreatic acinar cells [5,11,12]. Therefore, inhibiting ROS production or scavenging ROS may prevent development of acute pancreatitis by suppressing inflammatory cytokine expression in pancreatic acinar cells.

In the studies for the effect of DHA on Nrf2 activation, in human breast epithelial MCF-10A cells, DHA induced activation of PKCδ, phosphorylation of Nrf2, translocation of Nrf2 into the nucleus and its binding to antioxidant response element (ARE), and upregulated the expression of NQO1 and HO-1 [39]. 4-Hydroxy hexenal derived from DHA protected endothelial cells via Nrf2 activation and HO-1 induction using C57BL/6 Nrf2(+/+) or Nrf2(−/−) mice were fed a fish-oil diet for 3 weeks [40]. Treatment with DHA-derived cyclopentenones also increased DNA binding of Nrf2 and downstream expression of NQO1, similarly to the Nrf-2 activator sulforaphane [41]. Zhang et al. [42] showed that mice fed with a fish oil-enhanced diet demonstrated significant resistance to ischemia compared with mice fed with a regular diet. They showed that the protection was associated with HO-1 upregulation and Nrf2 activation in neuronal cells. In in vitro experiment, pretreatment of rat primary neurons with DHA significantly reduced neuronal death following oxygen-glucose deprivation with increased Nrf2 activation and HO-1 upregulation. These studies support the present finding showing that DHA triggered the nuclear accumulation of Nrf2 to induce transcription of Nrf2-mediated antioxidant genes *NQO1* and *HO-1* in AR42J cells following cerulein-stimulated oxidative stress.

Regarding the importance of Nrf2 signaling for preventing inflammation, NADPH oxidase-dependent ROS mediate amplified toll-like receptor 4 signaling and sepsis-induced mortality in Nrf2(−/−) mice [43]. Phosphorylation of IκB and cytokine expression were markedly higher in Nrf2-deficient macrophages than wild-type cells. In vivo studies showed greater lipopolysaccharide-induced pulmonary inflammation in Nrf2(−/−) mice as compared to Nrf2(+/+) mice. Kobayashi et al. [44] demonstrated that Nrf2 suppresses inflammatory response by blocking transcription of pro-inflammatory cytokines such as IL-6, IL-1β, and TNF-α in bone marrow-derived macrophages using Nrf2(−/−) mice and Nrf2(+/+) mice.

The alkaloid trigonelline can suppress Nrf2 expression in pancreatic cancer cells and promote cancer cell sensitivity to pro-apoptotic anticancer drugs [45]. In the present study, we validated the inhibitory effects of DHA-induced Nrf2 activation, *NQO1* and *HO-1* expression, ROS levels, and IL-6 expression in cerulein-stimulated pancreatic acinar cells by using the Nrf2 inhibitor trigonelline. Our results showed that trigonelline suppresses DHA-induced increase in *NQO1* and *HO-1* expression and thus, ROS levels and IL-6 expression in cerulein-stimulated AR42J cells. Thus, the effect of DHA on IL-6 expression is dependent on Nrf2 activation and its target antioxidant gene expression in pancreatic acinar cells.

Li et al. [46] demonstrated that the Nrf2-Keap1 signaling pathway mediates protective cellular responses to mitigate nitric oxide-induced damage and may contribute to the relative resistance of human colon carcinoma cells to nitric oxide-induced cytotoxicity. Park et al. [47] showed that HO-1 induction via Nrf2 activation may contribute to cytoprotection exerted by red ginseng extracts against oxidative stress in PC12 cells exposed to ubiquitous environmental contaminants such as polychlorinated biphenyls. These studies show that the antioxidant/cytoprotective enzymes including NQO1 and HO-1 are induced by oxidative stress and pathological conditions [41,42]. Lee and Surh [48] demonstrated that the effective strategy for chemoprevention is the blockade of DNA damage caused by carcinogenic insult. This can be achieved by stimulating detoxification of carcinogenic insult. Several ARE-regulated gene products such as NQO1 and HO-1 mediate detoxification and/or exert antioxidant functions thereby protecting cells from genotoxic damage. Therefore, selected Nrf2-Keap1-ARE activators, such as sulforaphane, curcumin, and caffeic acid, are potential chemopreventive agents. In the present study, we demonstrated a potential use of DHA as a chemotherapeutic agent by increasing detoxification/antioxidant enzymes.

Regarding the relation of HO-1 and development of acute pancreatitis, luteolin protects mice from severe acute pancreatitis by exerting HO-1-mediated anti-inflammatory and antioxidant effects, which was suppressed by ZnPP [49]. In addition, inhibition of NQO1 with dicumarol induces oxidative stress and inhibits pancreatic cancer cell growth [50]. In the present study, we confirmed the inhibitory effects of ZnPP, a HO-1-specific inhibitor, on DHA-induced upregulation of antioxidant enzymes NQO1 and HO-1 in cerulein-stimulated pancreatic acinar cells. We found that cerulein-stimulated ROS and IL-6 expression was reduced upon DHA treatment; however, this trend was reversed upon treatment with ZnPP. Therefore, DHA can exert positive modulatory effects on acute pancreatitis by inhibiting oxidative stress and inflammatory cytokine production by activating Nrf2 signaling to induce NQO1 and HO-1 expression in pancreatic acinar cells.

## 5. Conclusions

DHA enhances the antioxidant defense activities by upregulating the Nrf2 pathway to attenuate ROS levels and IL-6 expression in cerulein-stimulated AR42J cells. Therefore, DHA may prevent the development of oxidative stress-associated acute pancreatitis.
